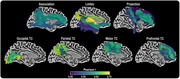# Evaluating white matter microstructure in aging and age‐related cognitive impairment: A comparison of free‐water and NODDI ISOVF diffusion models

**DOI:** 10.1002/alz70856_105013

**Published:** 2026-01-12

**Authors:** Aditi Sathe, Niranjana Shashikumar, Kimberly R. Pechman, Corey J Bolton, Shubhabrata Mukherjee, Seo‐Eun Choi, Brandon Klinedinst, Michael L. Lee, Phoebe Scollard, Emily H. Trittschuh, Panpan Zhang, Kurt Schilling, Bennett A. Landman, Shannon Risacher, Andrew J. Saykin, Paul K Crane, Timothy J. Hohman, Angela L. Jefferson, Derek Archer, Alzheimer's Disease Neuroimaging Initiative (ADNI)

**Affiliations:** ^1^ Vanderbilt Memory & Alzheimer's Center, Vanderbilt University Medical Center, Nashville, TN, USA; ^2^ Vanderbilt Memory and Alzheimer's Center, Vanderbilt University School of Medicine, Nashville, TN, USA; ^3^ Vanderbilt Memory and Alzheimer's Center, Nashville, TN, USA; ^4^ Department of Medicine, University of Washington, Seattle, WA, USA; ^5^ Department of Psychiatry and Behavioral Sciences, University of Washington School of Medicine, Seattle, WA, USA; ^6^ VA Puget Sound Health Care System, Seattle, WA, USA; ^7^ Department of Biostatistics, Vanderbilt University Medical Center, Nashville, TN, USA; ^8^ Vanderbilt University Institute of Imaging Science, Vanderbilt University Medical Center, Nashville, TN, USA; ^9^ Department of Radiology & Radiological Sciences, Vanderbilt University Medical Center, Nashville, TN, USA; ^10^ Department of Computer Science, Vanderbilt University, Nashville, TN, USA; ^11^ Department of Biomedical Engineering, Vanderbilt University, Nashville, TN, USA; ^12^ Vanderbilt Brain Institute, Vanderbilt University Medical Center, Nashville, TN, USA; ^13^ Department of Electrical and Computer Engineering, Vanderbilt University, Nashville, TN, USA; ^14^ Department of Radiology and Radiological Sciences, Vanderbilt University Medical Center, Nashville, TN, USA; ^15^ Indiana Alzheimer's Disease Research Center, Indiana University School of Medicine, Indianapolis, IN, USA; ^16^ Department of Radiology and Imaging Sciences, Indiana University School of Medicine, Indianapolis, IN, USA; ^17^ Indiana Alzheimer's Disease Research Center, Indiana University School of Medicine, Indianapolis, IN, USA; ^18^ Center for Neuroimaging, Department of Radiology and Imaging Sciences, Indiana University School of Medicine, Indianapolis, IN, USA; ^19^ Department of General Internal Medicine, University of Washington School of Medicine, Seattle, WA, USA; ^20^ Vanderbilt Genetics Institute, Vanderbilt University Medical Center, Nashville, TN, USA; ^21^ Department of Neurology, Vanderbilt University Medical Center, Nashville, TN, USA; ^22^ Vanderbilt Memory and Alzheimer's Center, Vanderbilt University Medical Center, Nashville, TN, USA; ^23^ Department of Neurology, Vanderbilt Memory & Alzheimer's Center, Vanderbilt University Medical Center, Nashville, TN, USA

## Abstract

**Background:**

White matter (WM) abnormalities are prevalent in aging and neurodegenerative cognitive decline. This study compares single‐shell free‐water (FW) imaging and multi‐shell NODDI Isotropic Volume Fraction (ISOVF) in assessing WM microstructure and their associations with cognitive decline.

**Methods:**

FW and ISOVF were quantified across 48 white matter tracts in cognitively unimpaired (CU) and impaired (MCI and AD dementia) individuals from ADNI and VMAP. Multi‐shell dMRI data (b=1000, 2000 s/mm2) were collated from 139 ADNI‐3 participants (age=75.9 ± 6.7; 56.8% female; 92.1% NHW) and 437 VMAP participants (age=65.8 ± 9.3; 57.0% female; 78.0% NHW). Memory and executive function composites were harmonized using ComBat. Covariates included age, sex, years of education, race, clinical status, and ApoE‐ε4 positivity. Analyses included linear correlations between FW and ISOVF, group comparisons, and age modeling via general linear models. Linear regression examined associations between tract‐specific metrics and cognitive composites, with bootstrapped comparisons testing differences in R2adj values between FW and ISOVF models.

**Results:**

FW and ISOVF showed strong correlations across key white matter tracts (Figure 1), particularly in transcallosal (TC) pathways, with the highest correlations in the calcarine sulcus TC (r=0.784), lingual gyrus TC (r=0.780), and ILF (r=0.775). Group‐wise analyses revealed significant CU vs. MCI differences in all 48 tracts, most notably in the ILF (FW: *p* = 1.05×10‐15) and temporoparietal SLF (ISOVF: *p* = 4.36×10‐14). Age effects were significant across all 96 measures, with FW explaining marginally greater variance than ISOVF. The highest R2adj values were in the ILF for FW (54.82%) and ISOVF (47.73%). FW in the lateral (42.93%) and anterior (47.84%) orbital gyri TC best explained memory variance, while FW in the ILF (51.98%) and ISOVF in the inferior parietal lobule TC (51.59%) best explained executive function. Bootstrapped comparisons identified significant R2adj differences between FW and ISOVF in 10 tracts for memory and 14 tracts for executive function, including the ILF, SLF, UF, and superior temporal gyrus TC.

**Conclusion:**

Both bi‐tensor FW and NODDI ISOVF showed robust associations with age and cognitive performance, varying in magnitude and tract specificity. FW and ISOVF provided largely comparable insights, highlighting their complementary value for understanding white matter health in neurodegenerative research.